# E-WE thrombin, a protein C activator, reduces disease severity and spinal cord inflammation in relapsing-remitting murine experimental autoimmune encephalomyelitis

**DOI:** 10.21203/rs.3.rs-2802415/v1

**Published:** 2023-04-17

**Authors:** Norah G Verbout, Weiping Su, Peter Pham, Kelley Jordan, Tia CL Kohs, Erik I Tucker, Owen JT McCarty, Larry S Sherman

**Affiliations:** Aronora (United States); Oregon National Primate Research Center, Oregon Health & Science University; Oregon National Primate Research Center, Oregon Health & Science University; Oregon Health & Science University; Oregon Health & Science University; Aronora (United States); Oregon Health & Science University; Oregon National Primate Research Center, Oregon Health & Science University

**Keywords:** Animal model, Multiple sclerosis, Relapsing/remitting, Demyelination, Inflammation, Steroids, Thrombin

## Abstract

**Objective:**

Relapses in patients with relapsing-remitting multiple sclerosis (RRMS) are typically treated with high-dose corticosteroids including methylprednisolone. However, high-dose corticosteroids are associated with significant adverse effects, can increase the risk for other morbidities, and often do not impact disease course. Multiple mechanisms are proposed to contribute to acute relapses in RRMS patients, including neuroinflammation, fibrin formation and compromised blood vessel barrier function. The protein C activator, E-WE thrombin is a recombinant therapeutic in clinical development for its antithrombotic and cytoprotective properties, including protection of endothelial cell barrier function. In mice, treatment with E-WE thrombin reduced neuroinflammation and extracellular fibrin formation in myelin oligodendrocyte glycoprotein (MOG)-induced experimental autoimmune encephalomyelitis (EAE). We therefore tested the hypothesis that E-WE thrombin could reduce disease severity in a relapsing-remitting model of EAE.

**Methods:**

Female SJL mice were inoculated with proteolipid protein (PLP) peptide and treated with E-WE thrombin (25 μg/kg; iv) or vehicle at onset of detectable disease. In other experiments, E-WE thrombin was compared to methylprednisolone (100 mg/kg; iv) or the combination of both.

**Results:**

Compared to vehicle, administration of E-WE thrombin significantly improved disease severity of the initial attack and relapse and delayed onset of relapse as effectively as methylprednisolone. Both methylprednisolone and E-WE thrombin reduced demyelination and immune cell recruitment, and the combination of both treatments had an additive effect.

**Conclusion:**

The data presented herein demonstrate that E-WE thrombin is protective in mice with relapsing-remitting EAE, a widely used model of MS. Our data indicate that E-WE thrombin is as effective as high-dose methylprednisolone in improving disease score and may exert additional benefit when administered in combination. Taken together, these data suggest that E-WE thrombin may be an effective alternative to high-dose methylprednisolone for managing acute MS attacks.

## Introduction

In the U.S., approximately one million patients live with multiple sclerosis (MS), an autoimmune disease characterized by central nervous system (CNS) inflammation and demyelination.^1^ The pathogenesis of MS includes disruption of the blood brain barrier (BBB), destruction of myelin, and damage to axons and neurons, leading to both acute and chronic motor, sensory and cognitive deficits.

Approximately 85% of patients present with relapsing-remitting MS (RRMS), in which acute attacks of clinical symptoms occur episodically and at unpredictable intervals.^2^ While disease-modifying agents can reduce the frequency and duration of MS relapses, they can provoke^3^ or worsen existing autoimmune disease in some settings. Further, the majority of these therapies predispose patients to infection, drug-induced autoimmune complications and malignancy.^4^ Relapsing MS attacks, even in patients taking disease-modifying therapies, are acutely treated with high-dose methylprednisolone over a period of 3 to 5 days.^5,6^ Glucocorticoids have both anti-inflammatory and immunosuppressive actions and are used widely to treat multiple inflammatory and autoimmune conditions. However, high-dose corticosteroids have serious adverse effects that can include, among others, gastrointestinal bleeding, psychosis, and flare up of some infections.^7,8^ Side effects can include weight gain, metabolic syndrome, avascular necrosis, and osteopenia.^7,8^ In RRMS, relapses occur frequently and approximately 26% of the relapses have sub-optimal response to steroids.^9,10^ Patients who present with disease progression or magnetic resonance imaging activity after 12 months of therapy often receive alternative treatments.^11,12^ Other non-steroid treatments for acute attacks include adrenocorticotropin hormone, the use of which is limited by high cost and patient access. Plasmapheresis may also be considered as an alternative, but is limited by side effects including infection and clotting.^13^ Accordingly, patients in which high-dose steroids are contraindicated or ineffective have limited treatment options for acute relapsing MS attacks.

Disruption of the BBB is an early event in MS that is linked to acute relapse.^14^ BBB injury facilitates the leakage of blood proteins into the CNS, including the procoagulant mediators fibrinogen, tissue factor and protein C inhibitor.^15–17^ Fibrinogen and fibrin, the product of fibrinogen cleavage by the serine protease thrombin, are both present in MS CNS lesions. The presence of fibrin in MS CNS lesions^18,19^ is indicative of BBB injury, leakage of plasma into the extracellular matrix, and thrombin activity in the CNS. Early studies in MS patients found that inhibiting thrombin and fibrin formation with heparin treatment reduced the severity of MS exacerbations,^20,21^ suggesting that anticoagulation may have therapeutic benefit. The potential bleeding complications associated with currently available anticoagulants present some risks and limitations to their use for MS.^22^ To date, randomized, controlled clinical studies have not been conducted to evaluate the potential benefits of anticoagulation in MS.

In murine experimental autoimmune encephalomyelitis (EAE), systemic anticoagulation with activated protein C (APC), hirudin, heparin, or fibrinogen depletion reduces disease severity and CNS inflammation,^16,23−27^ suggesting that extracellular matrix thrombin generation and fibrin may have pathogenic roles in MS. Endogenous APC is a vital endothelium-associated enzyme that anticoagulates the boundary layer of blood by degrading coagulation factors Va and VIIIa.^28^ In addition, APC induces antiapoptotic and anti-inflammatory signaling,^29^ as well as stabilizes endothelial barriers *in vitro* and *in vivo*.^30^ Systemically infused recombinant APC improves outcomes in EAE,^16,31^ as well as experimental stroke, traumatic brain injury, amyotrophic lateral sclerosis and Alzheimer’s disease.^32–38^

E-WE thrombin is a recombinant thrombin analog with two specific amino acid mutations (W215A/E217A) that generate an enzyme with significantly reduced procoagulant activity.^39^ Compared to thrombin, the enzymatic activity of E-WE thrombin towards fibrinogen and platelet protease activated receptor-1 (PAR1) is reduced 19,000 and 1,200-fold, respectively.^40^ Importantly, E-WE thrombin retains 10% of the antiinflammatory and anticoagulant functions of thrombin when complexed to the endothelial receptor thrombomodulin.^41^ Due to this re-design of the molecule, E-WE thrombin selectively activates protein C, which is complexed on cell surfaces with the endothelial protein C receptor (EPCR), to form APC.^42^ Of particular relevance to MS, E-WE thrombin conveys therapeutic benefit in the pathogenesis of inflammatory disease. In a model of inflammatory joint disease, daily treatment with E-WE thrombin after disease onset suppressed collagen-induced arthritis in mice^43^ illustrating the potential utility of E-WE thrombin in treating other immune diseases, including MS.

In animal models, E-WE thrombin inhibits thrombus growth without systemic anticoagulation or hemostasis impairment.^44–47^ E-WE thrombin has been found to be safe and well tolerated in a phase 1 evaluation in healthy adult volunteers^47^ and in a phase 2 study of patients with renal failure (NCT03963895). Our published data indicate that administration of E-WE thrombin improves neurologic outcome and attenuates CNS damage in myelin oligodendrocyte glycoprotein (MOG) experimental autoimmune encephalomyelitis (EAE).^48^ Here we evaluate the therapeutic potential of E-WE thrombin as an alternative to high-dose steroids for severe RRMS attack.

## Materials And Methods

### Materials

*E. coli* expressing prethrombin-2 containing the W215A/E217A active site mutations (E-WE thrombin) was generated by using site-directed mutagenesis and an established *E. coli* expression system as previously described.^47^ The prethrombin-2 zymogen was activated using ecarin (Pentapharm), followed by chromatography steps to purify the E-WE thrombin active enzyme, and concentration and diafiltration into storage buffer for injection.

### Animals

Wild-type female mice were housed in the Small Laboratory Animal Unit at the Oregon National Primate Research Center (ONPRC), Oregon Health & Science University. The study was conducted in accordance with National Institutes of Health Guidelines for the use of experimental animals, and the protocols were approved by the ONPRC Institutional Animal Care and Use Committee.

### Induction of EAE and Clinical Scoring

SJL mice were inoculated subcutaneously with a 0.2 mL emulsion containing 150 μg proteolipid protein (PLP) peptide 139–151 (Peptides International, Louisville, KY) in saline and equal volume of complete Freund’s adjuvant (CFA) (Difco Laboratories, Detroit, MI) containing 200 μg heat inactivated Mycobacterium tuberculosis H37RA (Difco Laboratories, Detroit, MI) and monitored for clinical symptoms as previously described.^49^

Neurologic impairment was scored according to the following scale: 0, normal; 1, limp tail or mild hind limb weakness; 2, moderate hind limb weakness or mild ataxia; 3, moderately severe hind limb weakness; 4, severe hind limb weakness or mild forelimb weakness or moderate ataxia; 5, paraplegia with no more than moderate forelimb weakness; and 6, paraplegia with severe forelimb weakness or severe ataxia or moribund condition.^50^ Mice were monitored for changes in disease score up to day 29. At the end of the experiment, animals were euthanized and tissues harvested for histopathological analyses. In accordance with the local animal ethics guidelines, mice were euthanized once a score of 5 was observed.

### Treatments

E-WE thrombin was administered intravenously at a dose of 25 μg/kg every other day once a clinical score of 2 was observed (typically at day 11 post-inoculation). Vehicle control was administered in the same manner. Methylprednisolone (Sigma-Aldrich) was administered intravenously at a dose of 100 mg/kg on one occasion once mice reached a clinical score of 2. Mice received experimental treatments through the lateral veins in their tails.

### Experimental groups

In these studies, the following experimental groups were defined to evaluate the different experimental conditions examined:

Design 1:Mice were divided into two groups and at the onset of the initial attack, dosed with E-WE thrombin or vehicle every other day, for a total of 4 doses (Days 11, 13, 15, 17). At onset of relapse, mice received E-WE thrombin or vehicle for a total of 4 doses (Days 21, 23, 25, 27).
Design 2: Mice were divided into two groups and at the onset of the initial attack, dosed with E-WE thrombin or vehicle every other day, for a total of 4 doses (e.g. days 11, 13, 15, 17). Mice were observed through relapse without additional treatment.
Design 3: Mice were divided into four groups and at the onset of the initial attack, dosed as follows. In the first group, mice received E-WE thrombin administered every other day, for a total of 4 doses (e.g. days 11, 13, 15, 17). In the second group, mice received intravenous doses of vehicle, administered every other day, for a total of 4 doses (e.g. days 11, 13, 15, 17). In the third group, mice received a single intravenous dose of methylprednisolone upon reaching a clinical score of 2. In the fourth group, mice received a single intravenous dose of methylprednisolone upon reaching a clinical score of 2 and E-WE thrombin administered every other day, for a total of 4 doses (e.g. days 11, 13, 15, 17).

The dose level and dosing regimen for E-WE thrombin were based on previous studies.^43,48,51^ For methylprednisolone, the dose level selected was based on a previous study.^52^

### Immunohistochemistry

Spinal cords were dissected from the spinal columns and paraffin sections from the lumbar region were prepared and analyzed for changes in the expression of CD45, CD3, and myelin basic protein (MBP) using multi-channel fluorescence immunohistochemistry using immunostaining methods as previously described.^50^ Rabbit-anti CD45 (1:50, Abcam), rat anti-CD3 (1:100, Abcam) and mouse anti-MBP (1:1000; Covance) and appropriate fluoroconjugated secondary antibodies (Alexa 546 or Alexa 488, Molecular Probes, Inc.) were used. Cell nuclei were visualized by staining with DAPI (1:5000, Molecular Probes, Inc.). Sections were photographed with an Axioskop 40 fluorescence microscope (Zeiss) and images processed under identical conditions using Image J (version 1.53t).

### Statistics

Disease score data are expressed as the means ± SEM. For experiments in which two treatment groups were compared (vehicle, E-WE thrombin), statistical differences in average disease score between treatment groups were evaluated by 2-way ANOVA and cumulative disease index scores were evaluated by Mann-Whitney. For experiments in which four treatment groups were compared (vehicle, E-WE thrombin, methylprednisolone, E-WE thrombin + methylprednisolone), statistical differences in average disease score were analyzed by repeated measures ANOVA and cumulative disease index scores were analyzed by 1-way ANOVA, using Dunnett’s Multiple Comparison test. A p value of less than 0.05 was considered significant. For immunohistochemistry, data are expressed as the means ± SD and were analyzed for significance using a one-way analysis of variance (ANOVA) followed by Bonferroni’s Multiple Comparison test. A value of p < 0.05 was considered significant. Statistical analyses were made using GraphPad Prism (version 5.02, GraphPad Software, San Diego, CA).

## Results

### E-WE thrombin reduces disease severity in relapsing-remitting EAE

**Figure 1:**
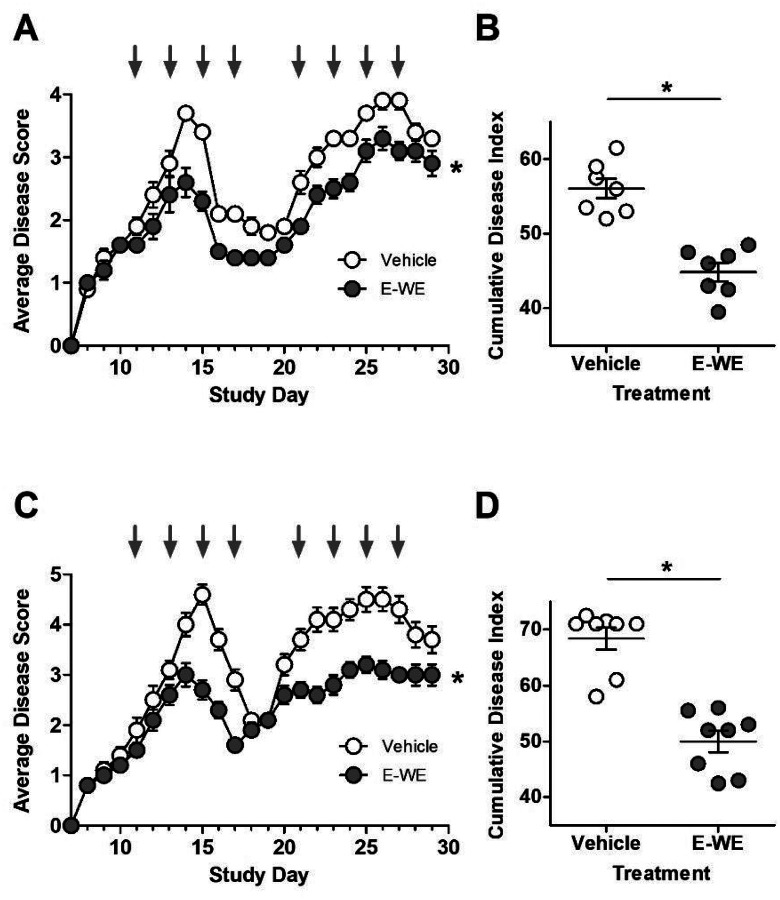
Administration of E-WE thrombin during attack and relapse reduces disease severity in relapsing remitting EAE. Female SJL mice (n = 7–8/group) were inoculated with a 0.2 mL emulsion (sc) containing 150 μg PLP 139–151 in saline and equal volume of CFA containing 200 μg of Mycobacterium tuberculosis H37RA. Once a clinical score of 2 was observed, mice were administered E-WE thrombin (25 μg/kg; iv) or vehicle every other day for four days at the first attack and again during relapse. Shown are average disease scores (± SEM) and cumulative disease score (± SEM) for two separate experiments (A, B) and (C, D). Statistical differences between treatment group for average disease score were evaluated by 2-way ANOVA, p < 0.05, *compared to vehicle. Cumulative disease index was analyzed by Mann-Whitney, p < 0.05, *compared to vehicle.

In our first set of experiments, we evaluated the ability of E-WE thrombin to improve neurologic impairment compared to vehicle control when administered at the onset of symptoms of both the initial attack and at relapse. Compared with vehicle, mice treated with E-WE thrombin had significantly lower disease scores following the initial onset of disease and during the relapse (Fig. 1A, C). Cumulative disease indices in two independent experiments demonstrated significant reductions in disease burden (Fig. 1B, D).

**Figure 2:**
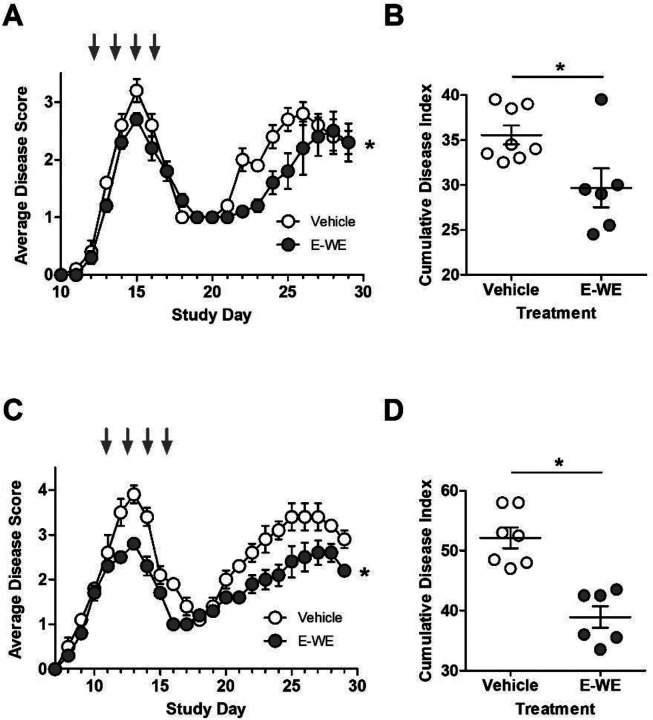
E-WE thrombin delays relapse onset and attenuates disease severity. Female SJL mice (n = 6–8/group) were inoculated with a 0.2 mL emulsion (sc) containing 150 μg PLP 139–151 in saline and equal volume of CFA containing 200 μg of Mycobacterium tuberculosis H37RA. Once a clinical score of 2 was observed, mice were administered E-WE thrombin (25 μg/kg; iv) or vehicle every other day for four days at the first attack and assessed without additional treatment during relapse. Shown are the average disease scores (± SEM) and cumulative disease score (± SEM) for two separate experiments (A, B) and (C, D). Statistical differences between treatment group for average disease score were evaluated by 2-way ANOVA, p < 0.05, *compared to vehicle. Cumulative disease index was analyzed by Mann-Whitney, p < 0.05, *compared to vehicle.

We next evaluated whether E-WE thrombin administered only during the initial attack could delay or reduce disease severity of the relapse. In two separate experiments, we found that dosing during the initial attack reduced disease scores during the initial attack and also delayed the onset of relapse and the severity of clinical disease scores during relapse (Fig. 2A, C). In these experiments, treatment with E-WE thrombin only during the initial attack led to a significant reduction in the cumulative disease index that was comparable to animals treated during both the initial attacks and during relapses (Fig. 2B, D).

### E-WE thrombin demonstrates comparable improvements in disease severity to methylprednisolone

**Figure 3:**
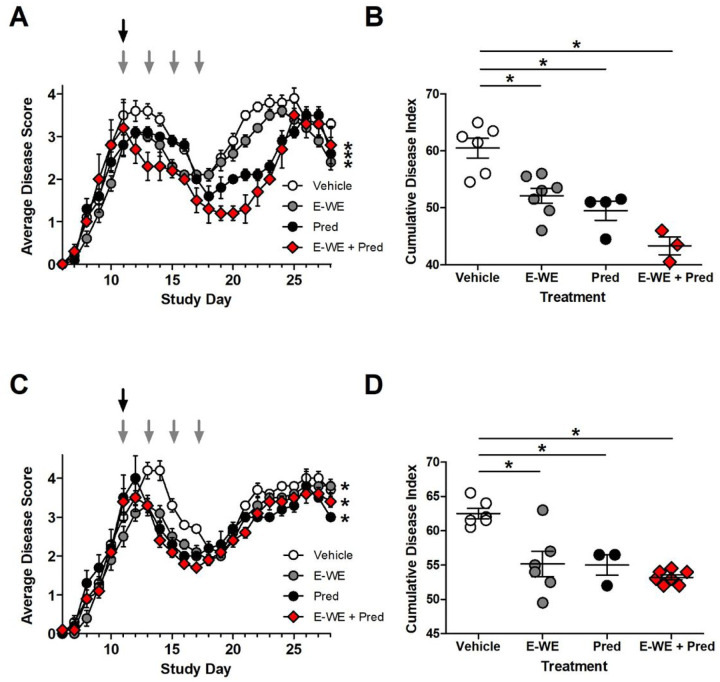
Effect of E-WE thrombin in methylprednisolone-treated SJL mice. Female SJL mice were inoculated as described above and treated once a clinical score of 2 was observed. Four treatment groups were evaluated: vehicle, E-WE thrombin (E-WE)(25 μg/kg; iv), methylprednisolone (MP)(100 mg/kg; iv) or the combination of E-WE thrombin and methylprednisolone (E-WE + MP). For methylprednisolone, a single dose was administered (black arrow) and for E-WE thrombin or vehicle, mice were dosed every other day for four days (gray arrows). Data are the means ± SEM of two separate experiments (A, B) and (C, D), n=3–7 mice/group. Statistical differences in average disease score were analyzed by repeated measures ANOVA, p < 0.05,* compared to vehicle. Cumulative disease index was analyzed by 1-way ANOVA, using Dunnett’s Multiple Comparison test, p < 0.05, *compared to vehicle.

Since high dose corticosteroids are the standard of care for managing acute MS relapse symptoms, we compared the effect of E-WE thrombin to methylprednisolone. In this third experimental design, mice were treated with either vehicle, E-WE thrombin, methylprednisolone, or the combination of both. Compared to vehicle, both E-WE thrombin and methylprednisolone significantly improved disease scores (Fig. 3A, C). The combination of E-WE thrombin with methylprednisolone significantly improved disease scores and the cumulative disease index (Fig. 3B, D) relative to vehicle control, but was not statistically different from either treatment administered alone, although there was a trend towards an additive effect in one of the experiments (Fig. 3B).

### E-WE thrombin reduces immune cell infiltration into the spinal cord

**Figure 4:**
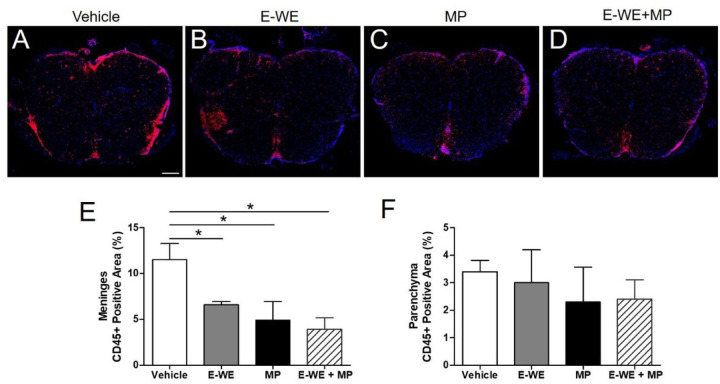
E-WE thrombin reduces CD45+ immune cell infiltration in the lumbar spinal cord sections. Lumbar spinal cord sections collected from mice on Day 29 were fixed, embedded in paraffin and immunostained for CD45+ cells (red). Cell nuclei were visualized by staining with DAPI (blue). Representative sections are portrayed for each treatment group (A-D). Magnification bar is 200 microns. The number of CD45 positive cells were quantified in the (E) meninges and (F) parenchyma using Image J. Data are the means ± SD of two experiments, n = 6–10 mice/group. Statistical differences in treatment group were analyzed by 1-way ANOVA, using Bonferroni’s Multiple Comparison test, p < 0.05, *compared to vehicle.

EAE severity is associated with immune cell infiltration into the CNS, therefore we next evaluated the ability of E-WE thrombin to reduce inflammatory cell accumulation in lumbar sections of the spinal cord. Since accumulation of CD3 + and CD45 + T cells are markers of CNS inflammation, we quantified the population of immune cells at the meninges and spinal cord parenchyma of the lumbar spinal cord following relapse (on day 29 post-inoculation) after treatment with vehicle, E-WE-thrombin (administered only during the initial attack as above), a single treatment of methylprednisolone, or E-WE-thrombin and methylprednisolone. Infiltration of CD45 + leukocytes into both the meninges and spinal cord parenchyma was significantly reduced in mice that received methylprednisolone, compared to vehicle (Fig. 4A), however E-WE thrombin alone did not reduce CD45 + recruitment to either the meninges or spinal cord parenchyma.

**Figure 5:**
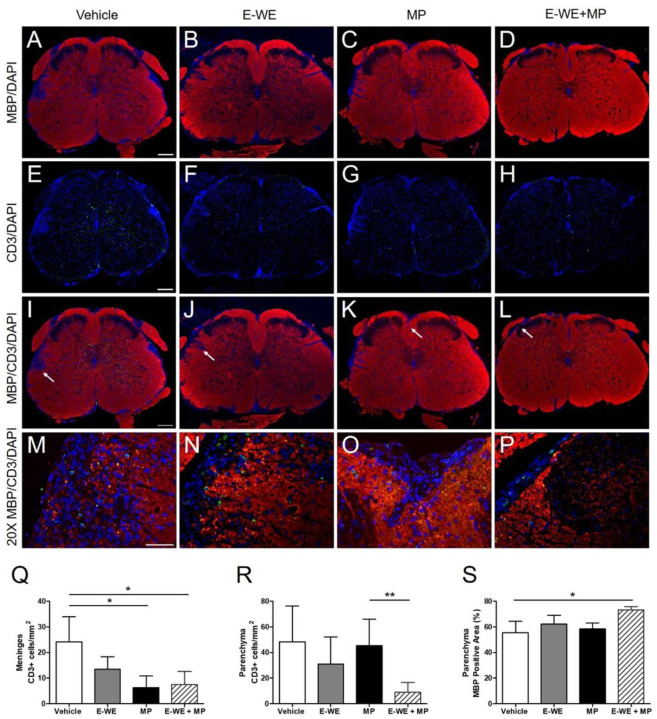
E-WE thrombin reduces CD3+ immune cell infiltration and improves MBP+ staining in lumbar spinal cord sections. Lumbar spinal cord sections collected from mice on Day 29 were fixed, embedded in paraffin and immunostained for MBP and CD3. Cell nuclei were visualized by staining with DAPI (blue). Representative photos for each treatment group are shown for MBP+ staining (A-D; red), CD3+ staining (E-H; green), the overlay of MBP+ and CD3+ at 5X magnification (I-L) and at 20X (M-P) magnification. In the third row, white arrows designate regions depicted at 20X magnification in the fourth row. Magnification bars are 200 microns (A, E, I) and 50 microns (M). The number of CD3 positive cells were quantified in the (Q) meninges and (R) parenchyma using Image J. The percentage of MBP positive area (S) was quantified using Image J. Data are the means ± SD of one experiment, n=3–4 mice/group. Statistical differences in treatment groups were analyzed by 1-way ANOVA, using Bonferroni’s Multiple Comparison test, p < 0.05, *compared to vehicle, **compared to methylprednisolone.

Compared to vehicle control, there was a significant reduction in the number of CD3 + cells around the meninges in mice treated with methylprednisolone (Fig. 5A). Treatment with E-WE thrombin appeared to reduce CD3 + cell infiltration but the effect was not statistically significant. The combination of E-WE thrombin with methylprednisolone was as effective as methylprednisolone alone. In the parenchyma, neither E-WE thrombin nor methylprednisolone appeared to reduce CD3 + populations compared to vehicle control, but the combination of both treatments appreciably reduced cell infiltration (Fig. 5A, C) compared to vehicle (p < 0.06) and significantly reduced infiltration compared to methylprednisolone alone.

### E-WE CNS disease pathology reduces thrombin

We examined the ability of E-WE thrombin to reduce demyelination in the lumbar spinal cord by evaluating myelin basic protein (MBP) staining after treatment with vehicle, E-WE-thrombin (administered only during the initial attack as above), a single treatment of methylprednisolone, or E-WE-thrombin and methylprednisolone. Compared to vehicle controls, neither E-WE thrombin nor prednisolone alone increased MBP + immunolabeling in the spinal cord white matter, however, MBP immunolabeling was significantly higher following the combination of both treatments (Fig. 5A, D).

## Discussion

In the present study, we evaluated the effect of repeat dose treatment with E-WE thrombin in a mouse model of relapsing remitting multiple sclerosis. Compared with vehicle control, treatment with E-WE thrombin attenuated neurologic impairment of both the initial attack and, with a second course of E-WE-thrombin, also reduced clinical scores during relapses. These findings are consistent with our previous work demonstrating that pharmacologic activation of endogenous protein C by E-WE thrombin^3^ can reduce disease severity in an EAE model of mild to moderate deficits following inoculation with myelin oligodendrocyte glycoprotein (MOG) peptide.

Remarkably, compared to mice that received vehicle, those that received E-WE thrombin only during their initial attack experienced less severe clinical symptoms during the initial attack and relapse. In addition, there appeared to be a delay in the onset of relapse in the E-WE thrombin treated group, compared to the vehicle group. These data suggest that just a single course of treatment has the potential to produce sustained effects beyond the initial treatment. Interestingly, E-WE thrombin was as effective as methylprednisolone at reducing disease scores in both the initial attack and relapse. When E-WE thrombin was administered in combination with methylprednisolone, there appeared to be a potential synergistic effect in the first round, as mice receiving both treatments had lower disease scores than single treatments alone, however, this effect was less apparent in the second round of experiments. One possible explanation for the difference in these two experiments is that the average disease scores were higher on the day of treatment, indicating that the mice may have had a higher disease burden when treatment was initiated.

When we evaluated the histopathological sections of the lumbar spinal cords, we noted methylprednisolone and E-WE thrombin differentially impacted immune cell infiltration and disease pathology. CD3 + cell infiltration into the spinal cord parenchyma was markedly reduced in mice that received both treatments, compared to either treatment alone. By comparison, CD3 + infiltrates in the meninges were significantly lower in mice receiving methylprednisolone, but there was no further reduction when mice received both treatments. In contrast, infiltration of CD45 + leukocytes into both the meninges and parenchyma was significantly reduced in mice that received methylprednisolone, compared to vehicle, while E-WE thrombin alone did not reduce CD45 + recruitment to either the meninges or parenchyma. Lastly, compared to vehicle treatment, methylprednisolone somewhat attenuated loss of MBP in the lumbar spinal cords. Surprisingly, we found that E-WE thrombin administered on top of methylprednisolone significantly improved the amount of MBP + staining in the parenchyma of spinal cords, compared to methylprednisolone alone. It is possible, therefore, that methylprednisolone and E-WE-thrombin may have combinatorial effects on different aspects of the processes underlying neuroinflammatory demyelinating disease.

It is conceivable that E-WE thrombin could influence remyelination by promoting the maturation of oligodendrocyte progenitor cells (OPCs) into myelinating oligodendrocytes. The PAR1 thrombin receptor is expressed by OPCs^53,54^ and thrombin can block OPC maturation in a PAR1-dependent manner.^55^

Furthermore, *Par1* gene deletion results in earlier onset of spinal cord myelination and treatment of OPCs with a PAR1 inhibitor enhanced OPC maturation into oligodendrocytes.^55^ Collectively, these findings suggest that thrombin accumulation in inflammatory demyelinating lesions can prevent OPC maturation and remyelination, and therapeutic strategies that downregulate thrombin activity, such as E-WE thrombin may overcome this pathway.

The findings described herein with the relapsing-remitting MS model are consistent with our previous evaluation in the MOG model, demonstrating that E-WE thrombin suppresses the progression of EAE at both the clinical and histopathological levels. The mechanism by which E-WE thrombin reduces clinical severity in EAE is presumed to be a consequence of endogenous generation of APC and the subsequent effects of APC. APC, the enzymatically active form of the zymogen protein C, is a serine protease at the center of the protein C pathway. APC exerts both anticoagulant and cell signaling activities that include anti-inflammatory, anti-apoptotic, and endothelial-stabilizing actions.^30^ While administration of APC has been shown to be neuroprotective in mice with relapsing-remitting EAE,^16^ it is not clear whether the anticoagulant properties of APC, i.e., the inactivation of clotting factors Va and VIIIa and reduced thrombin activity, contribute to neuroprotection. In our previous study, we observed that E-WE thrombin treatment reduced the accumulation of fibrin/fibrinogen in spinal cords. Leakage of fibrinogen into the spinal cords in EAE is known to activate microglia and participate in axonal damage,^56^ and inhibiting this pathway has been reported to reduce disease burden.^57^ However, in this study, we did not observe reduced fibrin/fibrinogen accumulation in histological sections of the spinal cord collected at the end of the relapse (not shown). This difference could be related to variations in the mouse strain or disease model, or possibly related to the time of evaluation (after first attack versus after relapse). The reductions in inflammatory cell recruitment we observed in the meninges and spinal cord parenchyma could be related to vascular protection or antiinflammatory effects.

Indeed, an APC variant which lacks antithrombotic activity but retains neuroprotective activity was found to be beneficial in a chronic progressive EAE model via suppression of microglial activity and stabilization of vascular injury.^31^ This same mutant was also found to confer benefit in disease models of ischemic stroke, traumatic brain injury, amyotrophic lateral sclerosis, and Alzheimer’s disease.^32,58,59^ The mechanistic basis for APC’s neuroprotective actions is hypothesized to be the result of cell signaling involving two G-protein-coupled receptors (GPCR’s): PAR1 and PAR3,^60^ which confer cytoprotective and antiinflammatory actions. Thus, it may be that cytoprotective and antiinflammatory signaling through PAR1 and PAR3 underlie the beneficial effects of E-WE thrombin on EAE progression and relapse. In agreement with this, we observed reduced leukocyte recruitment and higher myelin basic protein staining in mice treated with E-WE thrombin, compared to control.

In this study, sex as a biological variable was not evaluated since all experiments were conducted in female mice. While male mice can develop EAE, they often develop less severe symptoms and do not develop relapses.^61^ Thus, this factor precludes a conclusion whether E-WE thrombin would reduce clinical symptoms in male animals. Another limitation of this study is the restricted dose range. While we did evaluate two different dosing regimens, we did not assess whether augmenting the dose levels of E-WE thrombin would affect outcomes. Based on experience with this drug in other disease models^41,48,51,62^ and species,^47^ we believe that the dose level administered to mice was sufficient to produce the anticipated biological effect of endogenous APC generation. This was confirmed experimentally in a subset of mice that received the study drug. Following administration of E-WE thrombin, compared to control animals that did not receive study drug, we observed elevated levels of a standard laboratory marker of anticoagulation, the activated partial plasma time (aPTT) (not shown). Further, in an unpublished set of experiments in the MOG EAE model, we found that doubling the dose did not convey additional benefit on disease score.

In conclusion, E-WE thrombin has been shown to be protective in mice with EAE, a widely used model of MS that has been utilized to generate nearly all the approved drugs for patients with relapsing-remitting MS. Our data show that E-WE thrombin is as effective as high-dose corticosteroid treatment in improving clinical scores, and, when administered in combination with high-dose methylprednisolone, exerts additional benefit in relapsing-remitting EAE that exceeds either treatment alone.

## Data Availability

The datasets for this study are available upon request.
